# Skin marks in bottlenose dolphins (*Tursiops truncatus*) interacting with artisanal fishery in the central Mediterranean Sea

**DOI:** 10.1371/journal.pone.0211767

**Published:** 2019-02-05

**Authors:** Andrea Benedetto Leone, Giusy Bonanno Ferraro, Luigi Boitani, Monica Francesca Blasi

**Affiliations:** 1 Filicudi WildLife Conservation, Stimpagnato Filicudi, Lipari (ME), Italy; 2 Department of Biology and Biotechnologies, University “La Sapienza”, Rome, Italy; Aristotle University of Thessaloniki, GREECE

## Abstract

Skin marks occur frequently in many cetacean species across the globe revealing a broad spectrum of causes, including social interactions, infectious diseases and injuries produced by anthropogenic factors. The current study used photo-id data from 2005–2014 to estimate the skin mark pattern on resident bottlenose dolphins (*Tursiops truncatus*) from the Aeolian Archipelago (Italy). Thirteen skin mark types were identified and their origin, prevalence and permanence time were examined. The pattern of skin marks was assessed for the abundance, richness, distribution and severity in six body regions and compared among age classes, sex and degree of dolphins’ interaction with trammel nets (DIN). Our results showed higher prevalence, abundance, richness and distribution of skin marks in adults than in the younger age classes, with the exception of black marks and white ring lesions. The prevalence and abundance of skin marks were higher in males than females, with the exception of scratches and white patches. Moreover, gunshot wounds, mutilations and irregular dorsal fin edges were found only on adult males. Since males showed higher DIN than females and, in dolphins with higher DIN, skin marks were more abundant and frequently distributed in different body regions, the skin mark pattern in regard to DIN seems to be sex-related. The more severe marks were observed on adults, males and dolphins with higher DIN, namely skin disorder, tooth rake marks, small shallow indentations, deep indentations and mutilations. On the contrary, the severity of scratches, white patches and dark ring lesions was higher in females than males, but not significantly related to DIN and age of the individuals. Our results showed that photo-id data provide an efficient and cost-effective approach to document the occurrence of skin marks in free-ranging bottlenose dolphin populations, a critical step toward understanding the cause and supporting the conservation strategies.

## Introduction

Cetacean skin marks have been widely reported [1–3] and revealed a broad spectrum of causes, including infectious diseases (poxvirus and herpesvirus) [4–15] as well as environmental causes (solar radiation and water salinity) [16–19], injuries produced by sharks or parasitic copepods/diatoms, traumatic scarring [16, 20–25] and scars caused by propellers [16, 17, 19, 26, 27] or fishing gears [13, 28–31]. Biological and chemical contaminants may contribute to skin mark development in cetaceans [17, 19].

The skin mark pattern has been studied in bottlenose dolphins via photo-id techniques [11, 16, 19, 32–37]. In this species, natural tooth rake marks may be the result of intra-specific interactions among individuals with different sex and/or age for social purposes [35, 38]. Linear and curved scratches, notches and injuries may be caused by inter-specific interactions, such as the competition for food resources and habitat use or predator-prey relationship [23, 39–42]. A range of scarring features, such as the severity of epidermal marks [16], permanent white injuries [43], and tooth rakes [35], have been shown to vary with age/sex and can be assessed directly from photographs of dorsal fin or other body parts. Consequently, skin mark analyses may provide important information on the dolphin behaviour [44], the degree of interaction between individuals [45] and the determination of sex [36, 43] and age classes [16, 46, 47]. The intensity and amount of skin marks in dolphin populations may also reflect their general health status and the level of environmental/anthropogenic pressures in specific areas [17, 48, 49]. Consequently, assessing the skin mark pattern in certain dolphin populations might indicate changes in environmental conditions and in the exposure to pollutants and other negative anthropogenic factors.

Since 2005 a photo-id study was performed on an endangered small population of bottlenose dolphins in the Aeolian Archipelago (Sicily, Italy) [37, 45, 50]. The encounter rate of the dolphin groups has rapidly decreased during the last few years [37, 45, 50] and only a few individuals have been photo-identified in this area. In the Aeolian islands, the inshore occurrence of dolphins is mainly related to fishery activities [45] and, as fish stocks are generally declining, the individuals are increasingly competing with coastal artisanal fishing, including trammel nets [34, 51]. The dolphins may cause direct damage to nets by stealing fish from them, damaging and reducing the catch and disturbing the fishing operations [34]. Consequently, dolphin-fishery interactions can be dangerous, because they expose dolphins to the negative reaction of fishermen, who try to scare the animals away from the nets or kill them, often using harpoons or guns [34]. During the breeding season, females with calves show the strongest associations, spending more time in safer areas for feeding, foraging, resting or calf care/learning [37, 45, 50]. In contrast, males prefer areas where they have a high probability of locating and capturing the most appealing preys, such as those found in fishing areas and trammel nets [37, 45, 50]. Female and male groups are resident in the study area and some males specialized in trammel net foraging, forming small groups when depredating nets [45].

The current study used photo-id data to assess and quantify the types of skin marks in bottlenose dolphins interacting with artisanal trammel nets in the Aeolian Archipelago. Skin marks were identified and classified according to their origin, prevalence in the population and time of permanence in different body regions. The abundance, richness, distribution and severity of skin marks were also assessed and compared among groups of dolphins with different age, sex and degree of interaction with trammel nets.

## Materials and methods

### Survey and photo-id data

The study area covered 280 km^2^ of coastal area around Filicudi island, in the Aeolian Archipelago (Southern Tyrrhenian Sea, Italy—38°35' N, 14°34' E). Dedicated boat surveys were performed from June to September in 2005–2014. The surveys were carried out from 6.00 a.m. to 14.00 p.m., limited to sea states of Beaufort 3 or less and in good light conditions (visibility > 300 m) [37, 45, 50]. Bottlenose dolphins’ data were collected using a combination of focal group observations [52] with instantaneous data sampling [53] and photo-identification techniques applied to the natural markings of the dolphins’ bodies [54]. No specific permissions were required for these locations/activities under the Italian/regional regulations and field studies did not involve experimental manipulations of the dolphins. A ‘focal group’ was defined as the number of animals observed in an apparent association, moving in the same direction and often engaged in the same activity [52]. If a focal group split, a random sub-group was followed independently of group size and/or activity [55]. Each individual in a focal group was photographed and identified according to the natural markings on its body, especially on the dorsal fin, by standard photo-id techniques and videos taken during each sighting [37, 45]. Every three minutes, during each sighting, the presence of trammel nets within 100 meters from the focal group was also recorded. High-resolution photographs of distinctive dorsal fins were used to match dolphins with a photo-identification catalogue of known individuals [54]. The degree of interaction with trammel nets (DIN) of each dolphin in the catalogue was calculated as the percentage of three minutes periods when trammel nets were within 100 meters from the individual.

### Age class and sex estimation

As bottlenose dolphins are sexually dimorphic [43] and genetic samples were not collected, the sex of the individuals was determined primarily by opportunistic views of the genital region and was later verified by standard photo-id techniques and videos taken during each sighting. Additionally, the adult animals that had higher relative body length and were never observed in close contact with a calf during the study period were classified ‘estimated male’; all others were classified ‘estimated females’.

Age classes were attributed as follows [37, 46]: (1) adults are large and robust animals with a dark skin colour and many marks on the dorsal fin and body and, in case of females, are often accompanied by a calf; (2) juveniles are less-robust and smaller animals (at least two-thirds the length of adults), usually with less-distinctive nicks or without nicks on their dorsal fins and not obviously associated with an adult; (3) calves are smaller individuals often observed in close association with an adult [56] and usually without nicks on their dorsal fins.

### Classification of skin marks

**Fig 1:**
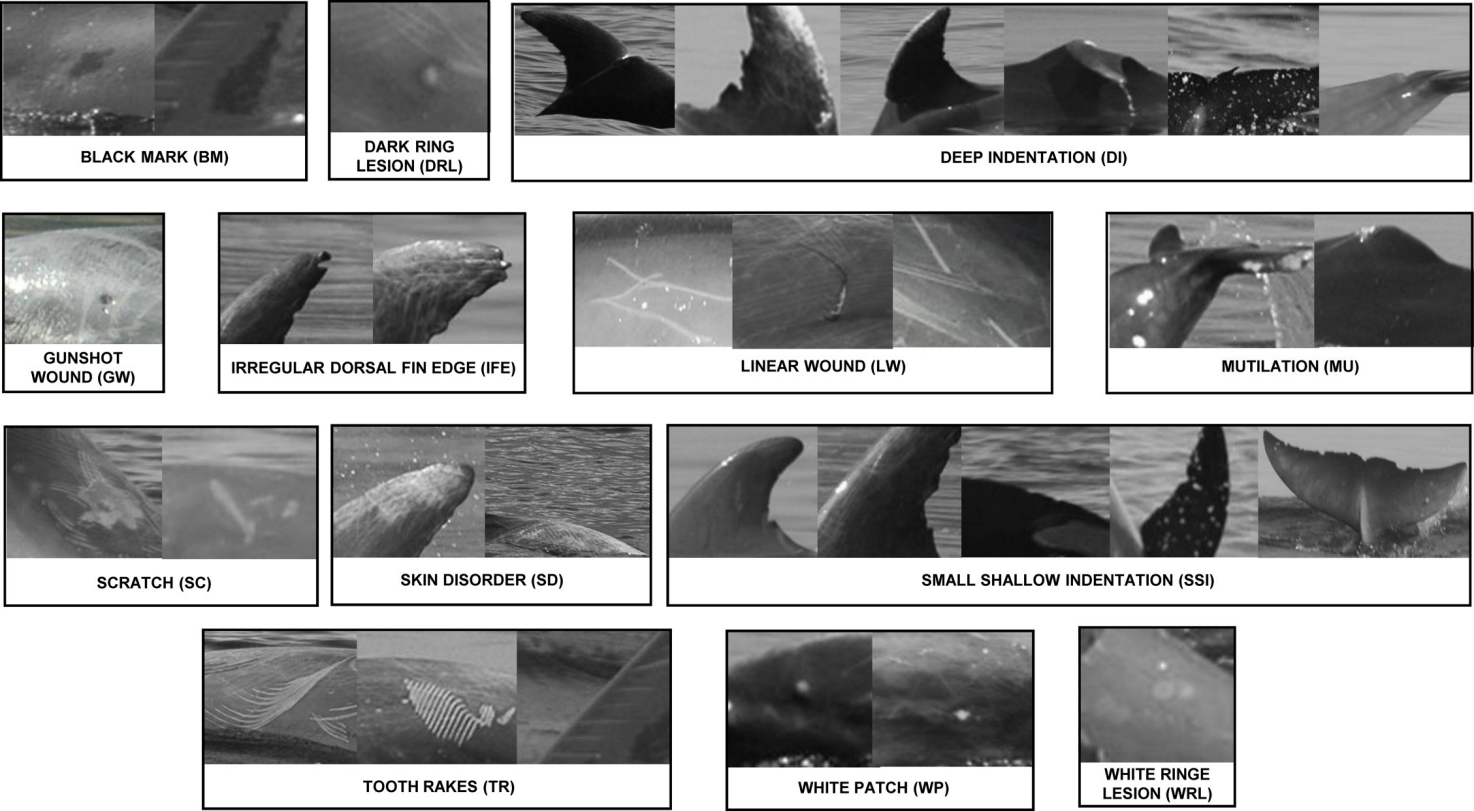
High-resolution photographs of the 13 skin mark types found in Aeolian bottlenose dolphins. Photographs show types of marks found on different body regions (dorsal fin, flanks, tail and flukes, back etc.) or with different degree of intensity.

For each photo-identified dolphin in the catalogue, high-resolution photographs of dorsal fin, right and left flanks, back, head, tail and flukes were visually screened for the detection of skin marks, and sorted according to standard protocols, using nicks, notches, scars, patches, injuries and other clear lesions or traumata. Skin marks were analysed and classified, according to previous data (Table 1 and Fig 1) and grouped together in one category if recorded on different body regions or with different degree of intensity (Fig 1).

**Table 1:** Skin marks types found in bottlenose dolphins from Aeolian Archipelago. The scientific papers that previously described skin marks and a brief description as these appeared in photographs are reported in the second and third columns of the table. Classification of skin marks types according to the origin, time of permanence on the dolphins’ bodies and their prevalence in the population (%) are also reported in the other columns.

*Skin mark*	*References*	*Description*	*Origin*	*Time of permanence*	*Prevalence (%)*
Black Mark (**BM**)	10; 16; 28; 63; 68–71	Small dark gray blemish with irregular rounded contour and lay flush with the rest of the skin, similar to 10; 16; 28; 68	Skin lesion	Permanent	19
Dark Ring Lesion (**DRL**)	7; 13; 16; 17; 19; 68–73	Pale areas of skin surrounded by a dark halo and most often circular [16]	Skin lesion	Temporary	32
Deep Indentation (**DI**)	13; 30; 31; 32; 62; 66; 70	Deep cut-like wound [32]	Traumata	Permanent	11
Gunshot Wound (**GW**)	13; 32	Permanent pit-like depression [32]	Traumata	Permanent	3
Irregular dorsal fin edge (**IFE**)	17; 62; 70; 71	Non-cleanly severed part of the dorsal fin with irregular borders [60]	Natural	Permanent	16
Linear Wound (**LW**)	7; 17; 29; 32; 36; 62; 66; 69–71; 74	Laceration in the epidermis, especially around the head, dorsal fin, flippers and fluke [62]	Traumata	Temporary	97
Mutilation (**MU**)	13; 16; 17; 29; 30; 32; 62; 66; 69; 74	It includes missing entire fluke or dorsal fin bent over [30]	Traumata	Permanent	5
Scratch (**SC**)	32; 63	No obvious permanent scar which include small nicks in the skin that do not penetrate the dermis [32]	Traumata	Temporary	81
Skin Disorder (**SD**)	13; 16; 71	White area composed of a mass of crisscrossed scratch marks and tooth rakes [16]	Traumata	Permanent	70
Small Shallow Indentation (**SSI**)	29; 32; 36; 62; 69–71	Half round or oval shaped cut [62]	Traumata	Permanent	84
Tooth Rakes (**TR**)	7; 17; 29; 32; 35; 36; 62; 63; 69–71	Parallel linear skin wounds or scars [60]	Traumata	Temporary	95
White Patch (**WP**)	13; 16; 17; 19; 28; 63; 68; 69–71	Small white patch slightly raised or lay flush with the rest of the skin [16]	Skin lesion	Temporary	54
White Ring Lesion (WRL)	16; 19; 70–72	Cream or white halo surrounding small circles of normally colored or black skin [16]	Skin lesion	Temporary	11

According to the descriptions in previous scientific papers, skin marks were also divided into: (1) Skin lesions, including marks due to infectious diseases such as bacterial, viral or parasitic infections [7, 10, 14, 16, 17, 19, 28, 57, 58]; (2) Traumata, including natural marks and scars due to intra- and/or interspecific interactions [7, 22, 35, 36, 41–44, 59–62], abrasions with rocks and other natural sharp objects while swimming [7, 44] and marks due to impact with vessels, propellers, interaction with fishing gears or direct damage by fishermen [13, 29, 30, 32, 62–67] (Table 1). Skin marks were also divided according to time of permanence: (1) Temporary, are skin marks with permanence time on the individual ≤ 4 years; (2) Permanent are skin marks with permanence time on the individual > 4 years or visible for the whole study period (Table 1).

### Skin marks analysis

In order to better describe the skin marks pattern according to age, sex, DIN, the “power” and the effect of every single mark on each individual, different parameters were calculated and analysed.

In particular, the prevalence and permanence time of each skin mark type were assessed for each individual. The prevalence was calculated as the number of dolphins with the mark type compared to the number of all marked and not marked individuals. Separately, the prevalence of each mark type was calculated according to (a) all marked individuals only (independently of the mark category), (b) all marked males/females/adults/juveniles and calves, (c) all individuals recorded with that mark type. The permanence was the time of persistence of each mark type on the animal’s body from the first year when it was visible as a fresh or old mark to the last year when it was not visible anymore. In details, the permanence time was calculated by analysing the photographs of different body regions for each individual and counting the number of years (from 0 to 10) when the mark type occurred. If the same mark type was found in different body regions or in more individuals, the longest time of permanence among them was retained for the analyses. A time of permanence on the body ≤ 4 years was classified as “temporary”, while a time of permanence > 4 years was classified as “permanent” (Table 1).

In order to assess if the skin marks pattern was related to age, sex and DIN, the abundance, richness and distribution of skin marks were also calculated. In particular, the abundance was the total number of skin marks (independently of the relative abundance of each type) among the marked individuals with at least one skin mark type; the richness was the number of skin mark types (from 0 to 13) recorded over the whole body of the individual; the distribution was the number of body regions (from 0 to 6: dorsal fin, head, flanks, back, tail and flukes) where each skin mark type was located. The abundance, richness and distribution were calculated for dolphins with different age class (adults/juveniles and calves) and for males and females separately. In addition, since the DIN was different for males and females, the abundance, richness and distribution were also calculated for males with DIN higher or lower than 40% and for females with DIN higher or lower than 20%. Normal distributions of parameters were checked using Shapiro-Wilk tests and the homogeneity of variances using Levene’s test. Kruskal-Wallis test was used to investigate the differences between these groups of data.

Finally, the severity of each mark type was calculated in order to estimate the degree of threat to the population. For each dolphin (a) “low” severity was assigned to benign marks, i.e. superficial, short and limited to a little part of the skin in all occurrences, (b) “high” severity was assigned to marks that were deeper, larger, found on several body locations and/or that could affect swimming, like a mutilation and (c) “medium” severity was assigned to marks with intermediate severity. In order to understand if more severe marks may affect specific groups of individuals, a Principal Component Analysis (PCA) was performed on the severity data and for distinct groups (males, females and different DIN). Kruskal-Wallis tests were conducted on the scores (new values of each dolphin on the principal components) of the leading principal components (e.g. explaining more than 60% of data variance). A score plot on the leading principal components was produced, representing each dolphin according to identified sex and DIN classes. The loadings, the Pearson correlation coefficients between the leading principal components and variables (i.e. severity of skin marks types), were used to identify correlations between marks and components as these were related to the variance explained by such a component. These analyses were performed using the software PAST.

## Results

Four hundred surveys were carried out over 564 hours for a total of 6.204 km surveyed from 2005 to 2014. A total of 185 sightings were recorded, corresponding to 120.55 hours spent with the dolphins (mean ± St. Dev. sighting time = 35.8 ± 33.4 minutes, range 10–194 minutes). The dolphin groups covered 1.320 km at an average (± St. Dev.) speed of 10.74 ± 2.45 km/h.

The data used in this study were obtained from digital photo-id photographs taken during 135 sightings.

Only good quality photographs were selected and analysed for photo-id (n = 5046) and the number of good quality photographs did not differ between early (n = 3042) and late summer (n = 2004) (Kruskal-Wallis, *p* = 0.001) or among years (Kruskal-Wallis, *p* = 0.01). In particular, photographs of dorsal fins were obtained for 38 dolphins (100%) while photographs of flanks (right and left indistinctly) for 97.4% (n = 37), of the head for 57.9% (n = 22) and of the tail, flukes and back for 52.6% (n = 20).

### Age class, sex estimation and DIN

According to the proportional body length, each individual was classified as: adult (n = 21, 55.3%), juvenile (n = 11, 28.9%) and calf (n = 4, 10.5%). For two dolphins (5.3%) age class was not determined. Of these individuals, 14 were classified males (36.8%; 12 adults and 2 juveniles) and 9 females (23.7%; 8 adults and 1 juvenile), while the remaining 15 dolphins (39.5%; 1 adult, 8 juveniles, 4 calves and 2 unclassified age) were not classified.

The DIN was estimated in adults and juveniles only. In particular, 29 dolphins (76.3%) were sighted in the proximity of trammel nets: for 27.6% of them (4 adults and 4 juveniles) the DIN was < 20%, while 34.5% (6 adults and 4 juveniles) had the DIN ≥ 40%.

In addition, the DIN was estimated for males and females separately. A DIN ≥ 40% was estimated in 50% of photo-id males (6 adults and 1 juvenile) and a DIN from 25% to 40% for the other males (6 adults and 1 juvenile). Five females (55.6%, 5 adults and 1 juvenile) had a DIN from 0–25% and 4 females (44.4%) a DIN over the 25%.

### Prevalence and permanence

Thirteen skin marks were identified and classified (Table 1) with various levels of prevalence in the population. In particular, tooth rake marks (TR), linear wounds (LW), scratches (SC), skin disorders (SD) and small shallow indentations (SSI) were found with the highest prevalence (over 60%) (Fig 1 and Table 1).

The skin mark types were classified in: (1) skin lesions (n = 4, 30.8%) or (2) traumata (n = 9, 69.2%) (Table 1). On the basis of the time of permanence on different body regions, the skin marks were distinguished as temporary (n = 6, 46.2%) or permanent (n = 7, 53.8%) marks (Table 1).

**Fig 2:**
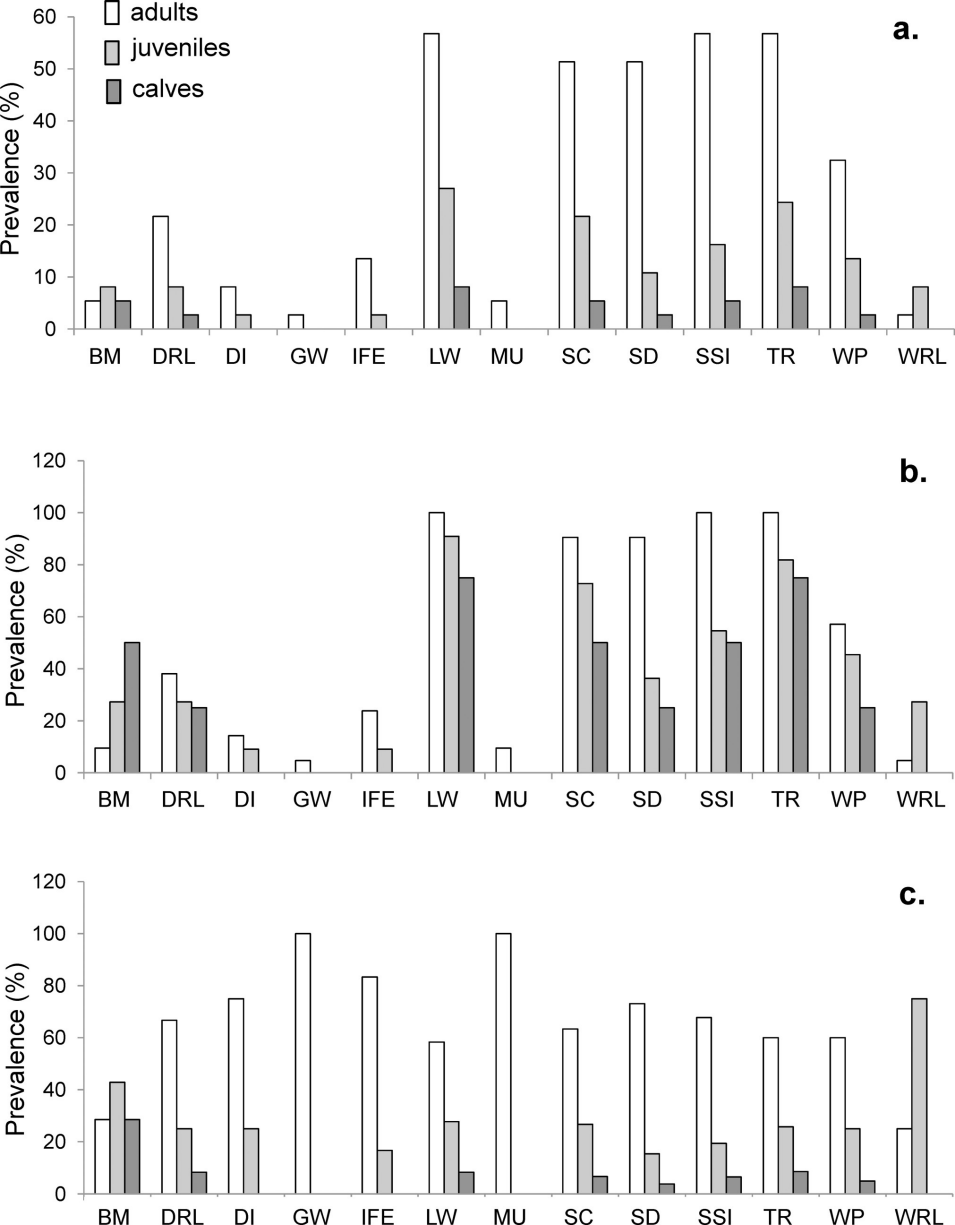
Prevalence (%) of skin mark types for adults, juveniles and calves. The prevalence was calculated as the number of adults, juveniles or calves with a skin mark type divided by a) the number of marked dolphins, b) the number of adults, juveniles or calves, respectively and c) the number of dolphins reporting that skin mark type. BM = Black Mark; DRL = Dark Ring Lesion; DI = Deep Indentation; GW = Gunshot Wound; IFE = Irregular dorsal Fin Edge; LW = Linear Wound; MU = Mutilation; SC = Scratch; SD = Skin Disorder; SSI = Small Shallow Indentation; TR = Tooth Rakes; WP = White Patch; WRL = White Ring Lesion.

**Fig 3:**
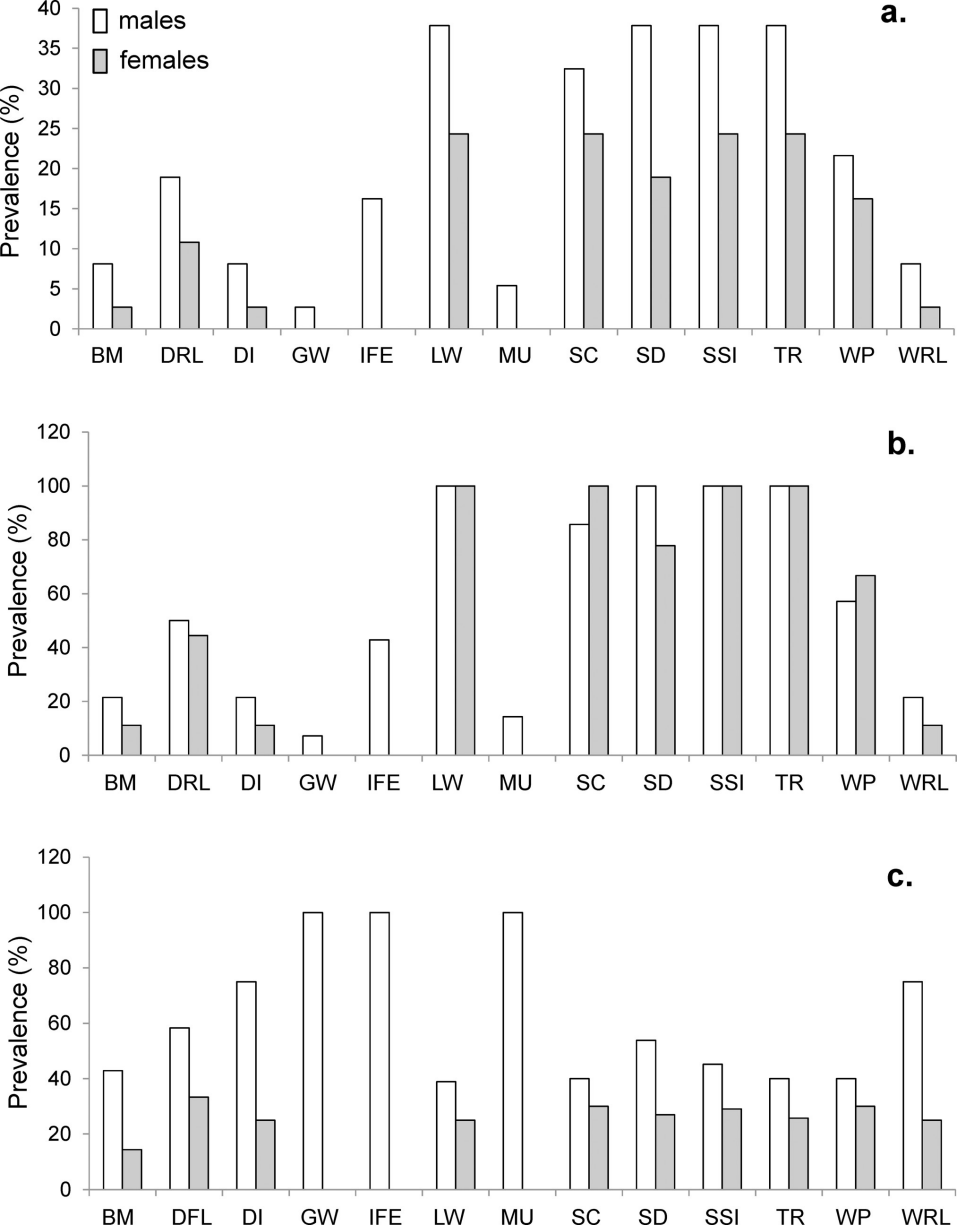
Prevalence (%) of skin mark types for males and females. The prevalence was calculated as the number of males or females with a skin mark type divided by a) the number of marked dolphins, b) the number of males or females separately and c) the number of dolphins reporting that skin mark type. BM = Black Mark; DRL = Dark Ring Lesion; DI = Deep Indentation; GW = Gunshot Wound; IFE = Irregular dorsal Fin Edge; LW = Linear Wound; MU = Mutilation; SC = Scratch; SD = Skin Disorder; SSI = Small Shallow Indentation; TR = Tooth Rakes; WP = White Patch; WRL = White Ring Lesion.

In order to analyse if the skin marks pattern was related to age and sex of the individuals, the prevalence (%) of each mark type was calculated for the three age classes (adults = 21, juveniles = 11 and calves = 4) (Fig 2) and for males (n = 14) and females (n = 9), respectively (Fig 3). Adults showed a higher prevalence of all marks than juveniles or calves, with the exception of black marks (BM) and white ring lesions (WRL) (Fig 2). Skin marks types LW, SC, SD, SSI, TR and white patches (WP) showed the highest prevalence values for all age classes among marked dolphins (Fig 2A), among adults, juveniles and calves separately (Fig 2B) and among dolphins with that skin mark (Fig 2C). In particular, 100% of the adults were injured by LW, SSI and TR marks (Fig 2B), representing over 50% of the dolphins with those marks on the whole population (Fig 2A), followed by SC and SD, over the 90% of adults. GW and MU marks were recorded only on adults (Fig 2).

The skin mark types LW, SC, SD, SSI, TR and WP had the highest prevalence in males and females among marked dolphins (Fig 3A), between males and females separately (Fig 3B) or among dolphins with that skin mark type (Fig 3C). However, higher prevalence of skin marks was reported in males (n = 14) than females (n = 9) (Fig 3B) with the exception of SC and WP that were found more frequently in females than males (Fig 3B). In particular, LW, SC, SD, SSI and TR marks were found on 100% of males, contributing 30% of marks found on the whole population (Fig 3A and 3B), while LW, SC, SSI and TR were found on 100% of females. Moreover, GW, IFE and MU marks were found only on male dolphins (Fig 3). Finally, SD, BM, DRL, DI and WRL were found more frequently in males than females (Fig 3B).

### Abundance, richness, and distribution

Abundance, richness and distribution for all groups of dolphins were not normally distributed (Shapiro-Wilk test, *p* < 0.05), but homogeneous (Levene's test, *p* > 0.05). In addition, adult dolphins were found to be more marked than the younger classes according to the Kruskal-Wallis tests (Table 2).

**Table 2:** Results of Kruskal-Wallis tests conducted on skin marks abundance, richness and distribution. The analyses were carried out for dolphins grouped according to age class (adults, juveniles and calves), sex (males and females) and degree of interaction with trammel nets (DIN). Since DIN values were higher in males (DIN from 25% to 56%, with 50% of males showing a DIN > 40%) than females (DIN from 0 to 36%, with 50% of females showing a DIN > 20%), a 40% threshold was used for males and 25% for females, respectively.

	*Abundance*	*Richness*	*Distribution*
**Adults vs Juveniles + Calves**	** **	** **	** **
Adults (n = 21)	11.86 ± 4.05	6.43 ± 1.86	3.58 ± 0.99
Juveniles + calves (n = 15)	7.93 ± 7.12	4.53 ± 2.83	2.46 ± 1.46
*Kruskal-Wallis test*	**0.01**	**0.02**	**0.01**
**Adults vs Juveniles**			
Adults (n = 21)	11.86 ± 4.05	6.43 ± 1.86	3.58 ± 0.99
Juveniles (n = 11)	8.82 ± 7.92	4.82 ± 3.03	2.43 ± 1.54
*Kruskal-Wallis test*	> 0.05	> 0.05	**0.04**
**Adults vs Calves**			
Adults (n = 21)	11.86 ± 4.05	6.43 ± 1.86	3.58 ± 0.99
Calves (n = 4)	5.50 ± 4.04	3.75 ± 2.36	2.17 ± 1.37
*Kruskal-Wallis test*	**0.02**	**0.04**	> 0.05
**Juveniles vs Calves**			
Juveniles (n = 11)	8.82 ± 7.92	6.43 ± 1.86	2.43 ± 1.54
Calves (n = 4)	5.50 ± 4.04	3.75 ± 2.36	2.17 ± 1.37
*Kruskal-Wallis test*	**0.05**	**0.05**	> 0.05
**Males vs Females**			
Males (n = 14)	14.64 ± 4.91	7.21 ± 2.15	4.01 ± 0.82
Females (n = 9)	10.89 ± 3.92	6.22 ± 1.48	3.38 ± 0.95
*Kruskal-Wallis test*	**0.05**	> 0.05	> 0.05
**Adult Males vs Adult Females**			
Adult Males (n = 12)	13.5 ± 4.03	6.92 ± 2.19	3.89 ± 0.82
Adult Females (n = 8)	10 ± 3.07	5.88 ± 1.13	3.35 ± 1.01
*Kruskal-Wallis test*	**0.04**	> 0.05	> 0.05
**DIN < 20% vs DIN ≥ 40% (only juveniles and adults)**			
Dolphins with DIN < 20 (n = 8)	7.62 ± 1.38	4.62 ± 0.73	2.72 ± 0.48
Dolphins with DIN **≥** 40 (n = 10)	11.50 ± 1.42	6.20 ± 0.64	3.78 ± 0.34
*Kruskal-Wallis test*	**0.05**	> 0.05	**0.05**
**Males DIN < 40% vs Males DIN ≥ 40% (only juveniles and adults)**			
Males with DIN < 40 (n = 7)	13.43 ± 1.40	8.00 ± 0.96	4.20 ± 0.30
Males with DIN ≥ 40 (n = 7)	17.16 ± 2.15	7.00 ± 0.65	4.04 ± 0.30
*Kruskal-Wallis test*	**0.05**	> 0.05	> 0.05
**Females DIN ≤ 25% vs Females DIN > 25% (only juveniles and adults)**			
Females with DIN ≤ 25 (n = 5)	11 ± 5	6.20 ± 1.92	3.49 ± 0.98
Females with DIN > 25 (n = 4)	10.75 ± 2.75	6.25 ± 0.96	3.25 ± 1.04
*Kruskal-Wallis test*	> 0.05	> 0.05	> 0.05

In particular, adults showed higher abundance, richness and distribution of marks than juveniles or calves; in adults and juveniles abundance and richness (but not the distribution) were higher than in calves; finally, only the distribution was higher in adults than juveniles (Table 2).

Skin mark abundance (but not richness and distribution) was higher in males than females as when considering only adult dolphins (Table 2). Finally, abundance and distribution were higher in dolphins with DIN ≥ 40% than dolphins with DIN < 20%. Grouping the dolphins for sex, males with DIN ≥ 40% showed higher abundance than those with DIN < 40% (Table 2). On the contrary, the abundance, richness and distribution did not differ in females with DIN higher or lower than 25% (Table 2).

### Severity

**Fig 4:**
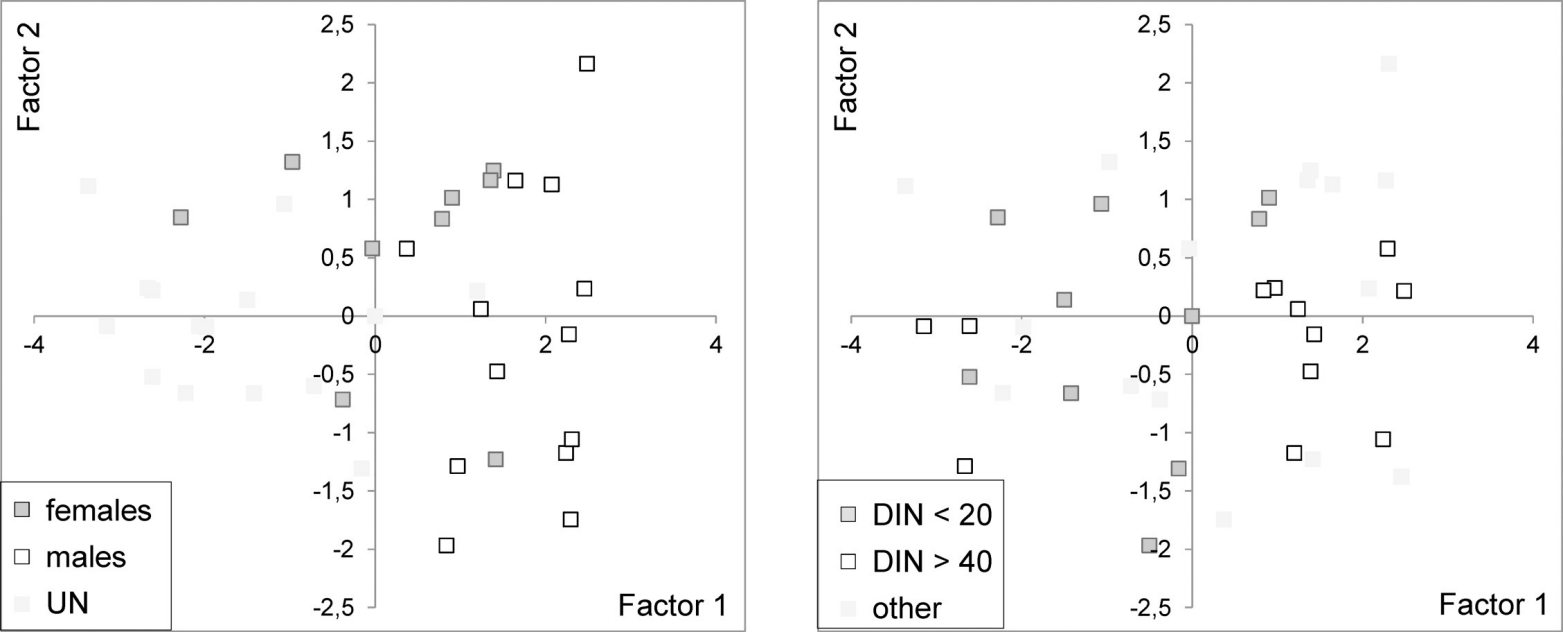
PCA on the severity of skin mark types. Plots of the component scores on Factor 1 and Factor 2 according to the percentages of explained variance showing different severity for marks between a) males and females and b) dolphins with DIN more than 40% or less than 20%. Each dolphin was identified by the photo-id code (PHD), sex (m = male, f = female, u = unknown), and age class (a = adult, j = juvenile, c = calf, u = unknown). For each mark type, the loadings on Factor 1 and Factor 2 were also reported.

The severity of the skin marks was found to be different in dolphins grouped by age, sex and DIN. In particular, two principal components explained about 60% of variance in the severity data (Factor 1 = 44.57% and Factor 2 = 13.18%). The scores on the leading principal components (Factor 1 and Factor 2) were normally distributed (Shapiro-Wilk, *p* > 0.01) and Levene’s test of homogeneity of variance showed no significant differences among distinct groups of dolphins (Levene’s test, *p* > 0.05). Both Factor 1 and Factor 2 were significant as for differences between males and females (Kruskal-Wallis test; Factor 1: Hc = 6.67, *p* = 0.009; Factor 2: Hc = 3.57, *p* = 0.05) and between adult males and adult females (Kruskal-Wallis test; Factor 1: Hc = 5.72, *p* = 0.01; Factor 2: Hc = 3.72, *p* = 0.05). Only Factor 1 was significant for differences among age classes (Kruskal-Wallis test, Hc = 9.39, *p* = 0.002), between dolphins with DIN more than 40% or less than 20% (Kruskal-Wallis test, Hc = 2.07, *p* = 0.01) and between adult dolphins with DIN more than 40% or less than 20% (Kruskal-Wallis test, Hc = 4.08, *p* = 0.004). On the Factor 1 vs Factor 2 score plot, each dolphin was represented according to identified sex and DIN threshold (Fig 4). In particular, the adult dolphins showed more severe marks than the other classes and males had more severe marks than females as well as adult males than adult females (Fig 4). Moreover, the dolphins (all or adults only) with DIN more than 40% showed more severe marks than dolphins with DIN lower than 20% (Fig 4).

The analysis of loadings showed the skin mark types more correlated with the leading principal components (Factor 1 and Factor 2) according to dolphins’ age, sex and DIN. In particular, the more severe marks for adult dolphins, males and dolphins with DIN > 40% were SD (loading on Factor 1 = 0.64), TR (loading on Factor 1 = 0.38), SC (loading on Factor 1 = 0.33), SSI (loading on Factor 1 = 0.27), DI (loading on Factor 1 = 0.13) and MU (loading on Factor 1 = 0.12) (Fig 4). On the contrary, the more severe marks for females were SC (loading on Factor 2 = 0.73), WP (loading on Factor 2 = 0.37) and DRL (loading on Factor 2 = 0.18), but they were not related to DIN and age of the individuals (Factor 2 was significant for sex only).

## Discussion

Epidermal marks are common in all bottlenose dolphin populations [13, 16, 19, 32, 62, 68] and other cetacean species across the globe [7, 29, 62, 69–75], but both their prevalence and severity may vary among populations as a result of environmental and anthropogenic factors and/or individual behaviour and metabolism [17, 19, 32, 63, 76–78].

In this study, thirteen mark types were identified in Aeolian bottlenose dolphins exposed to a wide range of natural and anthropogenic conditions, especially fishing activities. All skin marks were classified according to previous descriptions [16, 19, 32, 36, 62, 68, 71]. Of all the skin mark types, only 30.8% showed a clear natural cause, while 69.2% were associated with traumata. Two skin mark types (gunshot wounds and mutilations) were of anthropogenic origin, while the others might have caused by several factors or mixed causes. The prevalence of skin marks ranged from 3% to 97% (skin lesions: 11%-54%; traumata: 3%-97%) as already reported in other cetacean populations across the globe [16, 19, 29, 62, 71]. Although, the current study did not record tattoo skin disease, lobomycosis, candidiasis, herpesvirus, lunar and cloudy lesions, such as orange discolouration, deformities [13, 16, 19, 68] and shark bites [32, 62] as reported for some bottlenose dolphin populations or cetacean species from Atlantic and Pacific waters.

Our study showed higher prevalence in transient marks (from 11% to 97%; average prevalence = 61.7 ± 8.2%) compared to the permanent ones (from 3% to 84%; average prevalence = 29.7 ± 10.1%), with the exception of small shallow indentations (84%) and skin disorders (70%) [13, 16]. The majority of temporary marks were tooth rakes, linear wounds and scratches (prevalence > 60%), while ring lesions and white patches were less frequent (prevalence ≤ 50%) [73]. Tooth rake marks [21, 32, 35, 41–44, 61, 62, 71, 79] were found with prevalence values similar to those reported in bottlenose dolphins from Shark Bay, Australia (83% prevalence) [35]. On the other hand, linear wounds and scratches were found with higher prevalence values (97%) than those reported in other cetacean populations across the globe [7, 17, 29, 32, 36, 62, 66, 69–71], with the exception of the Mediterranean population of *Grampus griseus* (100% prevalence) [71]. As these marks are likely to result from parasites attachment (like *Pennella* spp.), rubbing with inanimate objects, fishing gears or rocky sea bottom or interacting with other species, such as fish and molluscs [7, 44, 69, 71], they may indicate a significant condition of distress for the Mediterranean cetaceans, but, as these marks have relatively short permanence time [7, 19, 29, 32, 35, 63, 72], they rarely pose a threat to the dolphins’ health and survival. In contrast, permanent marks have been associated to deep injuries from various sources [10, 16, 28–32, 36, 62, 68, 70, 79–81] and with different degree of severity [21, 22, 26, 32, 63]. In particular, permanent marks may lead to serious difficulties in physiological and behavioural patterns and, in some cases, to the death of the individual [66, 82]. For example, dolphins entangled in driftnets may be affected by injuries produced by fishermen, such as deep indentations or mutilations of stuck appendices [13, 29, 32, 62, 70], which might lead to dangerous infections and could limit swimming activities, inducing to a higher energy demand, starvation and consequent death. Mutilations, in our population, were reported in the dorsal fin and tail of 2 adult males with lethal consequences in one of them. On the other hand, a mark due to gunshot wound [13, 32, 83] was reported in the right flank of one adult male, suggesting that the interaction with trammel nets may be particularly dangerous in the Aeolian area.

Different studies analysed photo-identification data to estimate the prevalence of skin marks on cetacean populations in order to assess the causes of injuries or diseases [19, 28–32, 62, 63, 68–76, 82]. Some authors coupled photo-id techniques and histological analysis for a better understanding of marks’ aetiology, progress pattern and healing time [7, 10, 19, 72]. Some of the skin lesion types reported in this study (black marks, dark and white ring lesions and white patches) may have been caused by minor infections or parasites, possibly aggravated by pollution and/or by other negative environmental conditions [17, 19, 28, 36, 63, 71]. However, it is possible that the overfishing practices performed in bottlenose dolphins foraging areas might reduce the distribution of food resources, increasing the costs of feeding competition and affecting the animals’ health [37]. Other marks, such as irregular dorsal fin edge and deep indentations, might be due to a combination of behavioural and anthropogenic effects [62, 70, 71]. For example, skin disorder was very common in Aeolian bottlenose dolphins (70%) as well as small shallow indentations (84%), both resulting from social interactions and anthropogenic factors together [35–38, 60, 76, 79]. In particular, skin disorder showed a higher prevalence than that reported in the dorsal fin of Moray Firth bottlenose dolphins (28% of prevalence for pale lesions or abraded fin tips).

The prevalence of marks and marked dolphins were often used for demography studies [35, 36, 76–78], but, in many cases, only the dorsal fin was used in order to evaluate marks’ severity [16, 29, 35, 36, 79], while in our study different body regions were included to have a better representation of the skin marks’ pattern.

In this study, different parameters were used to assess the skin marks pattern. Marks’ prevalence and abundance based on population features were already reported for bottlenose dolphins in different areas [78] and for different species [69]. The severity of tooth rake marks was reported for two Scottish populations of bottlenose dolphins by using the overall rake direction, the average dorsal fin rake direction, the scarring percentage and the dorsal fin nick percentage [79]. Other authors [71] calculated the total number of marks and mark types (abundance) and the proportion of individuals with each mark (prevalence) grouping by age. In addition, these authors evaluated the mark change rate, e.g. the temporal variability of a mark type in terms of gain or loss rate [71]. However, in the current study, the distribution of skin marks was calculated, for the first time, as the percentage of coverage of a specific mark on six body regions [16, 79]. Moreover, the severity was established by an eye-evaluation of the intensity of a mark category in the different body regions and not as the coverage on the dorsal fin’s surface [17, 36]. This approach revealed to be very useful for the analyses of skin mark differences between age, sex and DIN classes.

The results of this study showed that the richness and distribution of skin marks were similar in both sexes and in the same body regions. On the other hand, males had more skin marks (higher prevalence) than females, with the exception of scratches and white patches. In particular, tooth rakes, linear wounds, skin disorders and small shallow indentations were higher in males than females, accounting for 30% of the total prevalence of marks in the population, and gunshot wounds, mutilations and irregular dorsal fin edge were found only in male individuals. These sex differences may be explained by variations in the behaviour among sexes [37, 45, 50], such as the more aggressive social behaviour [35–38, 60, 62, 76, 79] and/or the higher DIN of males compared to females [37, 45, 50]. Indeed, in this area, female bottlenose dolphins tend to prefer larger groups than males and to occur in safer areas for socializing, calving and calves cares and learning [37, 45, 50]. On the contrary, males prefer habitats where they could have a high probability of locating and capturing food resources, such as those found opportunistically in trammel nets [37, 45, 50]. Accordingly, in this study, the abundance and distribution of skin marks were higher in dolphins with DIN > 40% than those with DIN < 40%. Since only 4 females (44.4%) showed a DIN between 25% and 37%, but all photo-identified males (12 adults and 2 juveniles) showed a DIN > 25% (half of them even DIN > 40%), it is possible that the skin mark pattern in regard to DIN was sex-related. In particular, these males had higher abundance of marks and in more body regions (i.e. head, flanks, back and dorsal fin), suggesting that trammel net interactions might influence such a distribution pattern. In fact, among the 5 males with higher DIN (DIN > 80%), injuries by harpoons or gunfire and mutilations were recorded. On the contrary, the higher prevalence of scratch marks in females might be due to more "hit and run" interactions, both in social and predation activities [35] as already reported in other areas [32].

Previous studies on bottlenose dolphins and other species described an association between mark occurrence and fishery interaction and the prevalence of skin marks increased with increasing interactions [13, 29, 32, 62, 66, 74, 75]. In most cases, adults were more injured than the younger classes due to activities related to hunting and protecting calves [16, 35–37, 45, 50]. As expected [16, 19, 35, 36], skin marks abundance, richness and distribution were higher in adults than juveniles and calves, with the exception of black marks (reported in 5 juveniles/calves and only 2 adults) and white ring lesions (reported in 3 juveniles but missing in adults). In particular, more severe marks of natural or mixed causes were reported in adult dolphins, males and dolphins with DIN > 40%. On the other hand, the severity of scratches, dark and white ring lesions and white patches was higher in females than males, but not significantly related to DIN and age of the individuals. These differences may be due to potential infections and reduced immune system or healing processes of some individuals, such as pregnant/nursery females or calves [10, 16, 19, 28, 68, 69, 72, 73]. Some authors previously reported as male bottlenose dolphins from Shark Bay, Australia, may inflict deep tooth rake marks in females for sexual coercion, leading to epidermal infections and diseases [35] which, possibly, make the animals more vulnerable. It is not excluded that white patches and ring lesions are the result of skin infections from bacteria and viruses or derived from healing processes of several diseases [13, 17, 19, 28, 63, 68–71]. Histological analyses could provide in the future more information about the aetiology of such marks and the related risk for dolphins’ health in the study area.

In conclusion, this is the first work that analysed the skin marks pattern in bottlenose dolphins from one Mediterranean area using different parameters estimated from photo-identification data. The results of this study showed that the skin marks pattern of the Aeolian bottlenose dolphin population is strongly related to age, sex and degree of interaction with trammel nets. Our results also provided a new efficient and cost-effective approach to document the occurrence of skin marks in free-ranging populations, which is important to suggest cause-effect relationships with significant consequences in terms of conservation strategies [35, 43, 56, 77].
